# Prevalence and associated factors of mother-reported jaundice in newborns

**DOI:** 10.1590/1806-9282.20240691

**Published:** 2024-10-25

**Authors:** Arthur Cella, Juliana Coelho de Campos, Ícaro Colaiácovo, Gabriel Oscar Cremona-Parma, Eliane Traebert, Jefferson Traebert

**Affiliations:** 1 Universidade do Sul de Santa Catarina, School of Medicine – Palhoça (SC), Brazil.; 2 Universidade do Sul de Santa Catarina, Graduate Program in Health Sciences – Palhoça (SC), Brazil.

**Keywords:** Jaundice, Newborn, Child health, Prevalence

## Abstract

**OBJECTIVE:**

Newborns’ jaundice is the result of bilirubin accumulation as fetal hemoglobin is metabolized by the immature liver. This study aimed to estimate the prevalence of mother-reported newborn jaundice and associated factors.

**METHODS:**

A cross-sectional study was carried out using data from a longitudinal study involving 914 children. The data were reviewed using Poisson regression with a robust estimator in a hierarchical model in which the sociodemographic variables constituted the first level, those related to the conditions of the pregnancy constituted the second level, and those related to the report of jaundice, the third level. Prevalence ratios and their relevant confidence intervals were estimated.

**RESULTS:**

The prevalence of reported jaundice in newborns was 17.9%. The variables late pregnancy, urinary infection during pregnancy, and preterm and post-term birth were independently statistically associated with a higher prevalence of newborn jaundice reports.

**CONCLUSION:**

We can conclude that mother-reported newborn jaundice was 17.9% associated with maternal and birth aspects.

## INTRODUCTION

Hyperbilirubinemia, or jaundice, is a common benign condition in newborns and is one of the main causes of hospitalization in the first week of life^1,2^. Jaundice is observed in approximately 60% of full-term newborns and in 85% of premature newborns in their first week of life^1,3,4^.

Jaundice resulting from an increase in the indirect bilirubin fraction gives the skin a bright yellow or orange hue^5,6^. Conditions associated with direct hyperbilirubinemia give the skin a greenish-yellow color. However, these differences are only noticeable at very high levels of the pigment^5,6^.

In countries with few resources, kernicterus, defined as bilirubin encephalopathy that occurs when bilirubin serum levels are greater than 20 mg/dL in full-term neonates or lower in premature infants, continues to be an important and underestimated factor in neonatal morbidity and mortality^7^. In low- and middle-income countries, approximately 500,000 newborns per year develop extreme hyperbilirubinemia conditions (≥25 mg/dL), leading to approximately 114,000 neonatal deaths and 75,000 cases of kernicterus^7,8^. The main reason for this high morbidity and mortality is the inability to measure bilirubin levels adequately^8,9^.

Several papers have been published on neonatal jaundice, and a study published in the United States in 2018 revealed that the prevalence of neonatal jaundice was 55.2% of newborns. The study showed that 10% of black-skinned babies were diagnosed with jaundice observed due to hyperbilirubinemia but did not present with clinical jaundice^10^.

Family and healthcare professionals play an important role in the clinical diagnosis of early jaundice. A study of 1,666 mothers of babies with jaundice was carried out to assess mothers’ knowledge and practice regarding neonatal jaundice. The study showed that 77% of mothers had moderate to high knowledge about neonatal jaundice, demonstrating the relevance of mothers’ participation in the care of newborns with jaundice^11^.

Therefore, knowledge about the clinical evolution of these conditions and the presence of associated factors is essential for the adequate healthcare of newborns, as epidemiological characterization is extremely important so that primary and secondary care measures can be planned and carried out. This will enhance not only the reduction of jaundice prevalence but also the therapeutic and prognostic improvement. The objective of this study was to estimate the prevalence of mother-reported neonatal jaundice and identify associated factors in newborns.

## METHODS

This was an epidemiological study with a cross-sectional design using data from a longitudinal study called *Coorte Brasil Sul* ^12^ being developed in Palhoça, a municipality with approximately 220,000 inhabitants located in the metropolitan region of Florianópolis, SC. The study population was composed of children born in 2009, examined in 2015, and who are still being monitored. In the baseline study^12^, the following parameters were used to calculate the sample: population of 1,270 6-year-old children, anticipated prevalence of the outcome unknown (p=50%), 95% confidence level, and 2% relative error, which generated a minimum sample of 831 children. An additional number of children (10%) was added to compensate for any refusals, which yielded a final sample of 914 children randomly drawn from all 37 public schools and 19 private educational institutions in the municipality.

Data were collected through interviews in which mothers in their dwelling addresses were asked about their children’s prenatal, perinatal, and neonatal period descriptions. The team of investigators from *Coorte Brasil Sul* ^12^ was responsible for the data collection, together with the community health agents, all duly trained for the purpose.

In the present study, the dependent variable was the mother’s report on the occurrence of jaundice in the newborn, answering the question “*Did the newborn have jaundice/yellowing during the first 29 days of life?*” (yes/no). The independent variables were child’s gender; child’s skin color (categorized as white and non-white); mother’s education at the time of giving birth (categorized as up to 8 years of complete schooling and more than 8 years); mother’s age at child’s birth (categorized as less than 19, between 20 and 34, and greater than 35 years of age); number of prenatal visits (categorized as up to 6 and 7 or more); mode of delivery (vaginal or cesarean section); smoking, alcoholic beverages use, and use of illicit drugs during pregnancy (all, yes and no); occurrence of diabetes, hypertension, and infectious diseases during pregnancy (all, yes and no); preterm birth (categorized as up to 37 and 38 weeks or more); birth weight (categorized as up to 2,500 g and more than 2,500 g); birth weight by gestational age-GA (categorized as low for GA, adequate for GA, and high for GA); APGAR index in the first and fifth minutes (categorized as up to 7 and above 7); and head circumference (categorized as less than 32 cm, greater than 35 cm, and between 33 and 34 cm).

**Figure 1:**
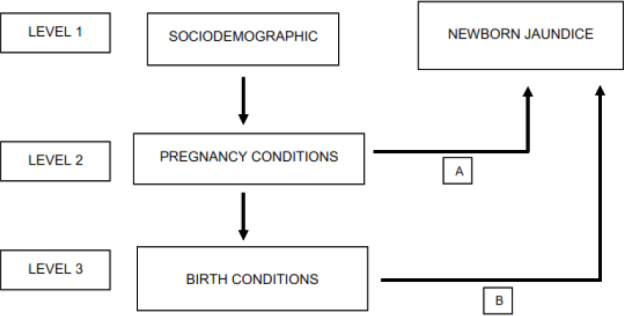
Hierarchical conceptual structure in blocks for reporting newborn jaundice.

The data were displayed in an Excel software spreadsheet and later exported to the SPSS Statistics for Windows software, version 18.0 (SPSS Inc., Chicago, IL, United States), where they were analyzed using Poisson regression with a robust estimator, hierarchical with the stepwise forward strategy. Prevalence ratios and their 95% confidence intervals were estimated. The hierarchical analysis model proposed for this study included three levels. The sociodemographic variables constituted the first level, the variables associated with pregnancy conditions constituted the second level, and the variables associated with the report of jaundice constituted the third level (Figure 1).

Initially, a bivariate analysis was carried out, with all the variables from each hierarchical level. A model was developed with the first-level variables that presented p<0.20. In this block, variables that presented p<0.05 were maintained. Subsequently, the second-level variables were added, which in the bivariate analysis presented p<0.20. At this point, the second-level variables that presented p>0.05 were removed from the model. The sociodemographic variables that presented statistical significance in the first stage of the multivariate model were maintained, regardless of the level of significance presented after the introduction of variables related to the conditions of the pregnancy. Thus, a two-level model was created. Subsequently, third-level variables were introduced, which in the bivariate analysis presented p<0.20. Variables from this third level that presented p<0.05 were maintained in the model without removing the variables from the previous levels. Thus, a three-level final model ensued.

This study complied with the ethical principles established in the Resolution of the National Health Council no. 466/2012 and was approved by the Research Ethics Committee of the *Universidade do Sul de Santa Catarina* under CAAE No.: 38240114.0.00005369.

## RESULTS

A total of 914 children were included in the study. The prevalence of mothers reporting newborn jaundice was 17.9% (95%CI 15.4; 20.3).

The results of the bivariate analysis showed that no variable from the first level (socioeconomic) was associated. The associated second-level variables (pregnancy conditions) were children born to mothers who had had a late pregnancy and had a 7.0% higher prevalence of jaundice reports (PR=1.07; 95%CI 1.01; 1.12) (p=0.013) compared to non-late pregnancy women. Urinary tract infection during pregnancy was associated with a 7.0% higher prevalence (PR=1.07; 95%CI 1.04; 1.11) (p<0.001). The occurrence of infectious diseases during pregnancy was also associated with a higher 6.0% prevalence (PR=1.06; 95%CI 1.02; 1.09) (p=0.001) compared to the non-occurrence of infectious diseases. As to the third level (birth conditions), preterm births had a 10.0% higher prevalence (PR=1.10; 95%CI 1.03; 1.17) (p=0.005), and post-term births had a 12.0% higher prevalence (PR=1.12; 95%CI 1.03; 1.22) (p=0.009) compared to those born at term. Children born weighing up to 2,500 g at birth had a 9.0% higher jaundice prevalence (PR=1.09; 95%CI 1.02; 1.16) (p=0.008) compared to those weighing more than 2,500 g (Table 1).

**Table 1:** Sociodemographic variables, pregnancy, and birth conditions.

Variables	Report of jaundice				
	n	%	PR_c_	95%CI	p
First Level—Sociodemographic					
Newborn gender					
Male	84	17.4	1.00		0.703
Female	84	18.4	1.01	0.98; 1.03	
Child’s skin color					
White	138	17.6	1.00		0.623
Non-white	30	19.2	1.01	0.97; 1.05	
Mother’s education at child’s birth					
Up to 8	68	16.6	1.00	0.98; 1.03	0.821
More than 8	82	17.2	1.00		
**Second Level—Pregnancy Conditions**					
Teenage pregnancy (age <19 years old)					
Yes	39	19.2	1.01	0.98; 1.05	0.561
No	126	17.4	1.00		
Late pregnancy (age 35 years or older)					
Yes	31	27.7	1.07	1.01; 1.12	0.013
No	134	16.4	1.00		
Number of prenatal visits					
Up to 5	10	14.3	0.98	0.94; 1.03	0.513
6 or more	132	17.1	1.00		
Smoking during pregnancy					
Yes	17	13.9	0.98	0.94; 1.01	0.201
No	146	18.3	1.00		
Urinary tract infection during pregnancy					
Yes	74	26.6	1.07	1.04; 1.11	<0.001
No	90	14.1	1.00		
Infectious diseases in pregnancy					
Yes	72	24.5	1.06	1.02; 1.09	0.001
No	87	14.5	1.00		
**Third Level—Birth Conditions**					
Delivery route					
Cesarean section	86	16.0	0.97	0.95; 1.01	0.077
Vaginal	132	17.1	1.00		
Gestational age					
Preterm (up to 37 weeks)	34	34.3	1.11	1.05; 1.18	<0.001
Post-term (42 weeks and a half)	5	11.4	1.14	1.06; 1.23	0.001
Term	112	16.0	1.00		
Birth weight					
Up to 2,500 g	24	32.0	1.09	1.02; 1.16	0.008
More than 2,500 g	144	16.7	1.00		
Weight by gestational age					
Large for gestational age	26	19.1	1.02	0.96; 1.09	0.483
Small for gestational age	19	23.2	1.04	0.98; 1.09	0.196
Suitable for gestational age	103	16.7	1.00		
Congenital anomalies					
Yes	7	26.9	1.05	0.95; 1.16	0.301
No	161	17.6	1.00		

95%CI: 95% confidence interval; PR_c_: crude prevalence ratio.

Significantly higher and independent prevalence of mothers reporting newborns jaundice indicated by the final hierarchical and adjusted model was due to late pregnancy with a 6% higher prevalence (PR=1.06; 95%CI 1.01; 1.12) (p=0.025); urinary infection during pregnancy with 13% higher prevalence (PR=1.13; 95%CI 1.06; 1.21) (p<0.001); preterm newborns with 10% higher prevalence (PR=1.10; 95%CI 1.03; 1.17) (p=0.005); and post-term newborns with 12% higher prevalence (PR=1.12; 95%CI 1.03; 1.17) (p=0.005) compared to full-term newborns (Table 2).

**Table 2:** Final hierarchical model for mother-reported jaundice in newborns.

Variables	Jaundice report		
	PR_a_	95%CI	p
First Level—Sociodemographic			
Late pregnancy			
Yes	1.06	1.01; 1.12	0.025
No	1.00		
**Second Level—Pregnancy Conditions**			
Urinary tract infection during pregnancy			
Yes	1.13	1.06; 1.21	<0.001
No	1.00		
**Third Level—Birth Conditions**			
Gestational age			
Preterm	1.10	1.03; 1.17	0.005
Post-term	1.12	1.03; 1.22	0.009
Term	1.00		

PR_a_: adjusted prevalence ratio; 95%CI: 95% confidence interval; p: p-value obtained by Poisson regression with robust estimator. Ominus test: p=0.977.

## DISCUSSION

Jaundice in newborns is a complex disease and has several determining factors that interact with its development, which is why the hierarchical analysis model of variables was used in the present study. After careful selection and analysis of the variables, it was found that late pregnancy, urinary tract infections during pregnancy, preterm newborns, and post-term newborns were independently associated with a greater prevalence of mother-reported jaundice in newborns. Among these variables, urinary infection during pregnancy had the highest magnitude of prevalence of mother-reported neonatal jaundice.

Urinary tract infection during pregnancy is a condition that showed a 13% greater prevalence of jaundice reports compared to mothers who did not experience this pathology. No direct relationship between gestational urinary tract infection and neonatal jaundice was found in the literature. However, there are studies that state that some drugs used to treat bacterial infections in the urinary tract, such as sulfonamides, can potentially cause harm in the case of lower bilirubin levels because they displace bilirubin from albumin, thereby increasing the free bilirubin fraction^13,14^.

Preterm newborns (<37 weeks of gestational age) have a 10% higher prevalence of jaundice occurrence compared to full-term newborns, which corroborates a study that places preterm birth as a risk factor for the development of neonatal jaundice^15^. This is mainly due to increased bilirubin production, immaturity in the uptake and conjugation of bilirubin, and increased enterohepatic circulation of bilirubin due to intestinal immaturity^15,16^. Out of the 125 newborns entered in the US voluntary registry of kernicterus, 24% were born preterm^17^. Preterm newborns have red blood cells with a shorter life cycle and, therefore, have relatively increased bilirubin production rates compared to healthy full-term newborns^18^.

It was observed that post-term newborns (>42 and a half weeks) had a 12% higher prevalence of jaundice than newborns at term. Post-term birth, as well as pre-term birth, are a risk factor for the development of neonatal jaundice^15,19^. On the contrary, the Guidelines of the American Academy of Pediatrics report that children born after 40 weeks or more of pregnancy and who were formula-fed have a very low risk of developing severe hyperbilirubinemia^20^.

In relation to maternal age, late pregnancy (over 35 years of age) was associated with a 6% higher prevalence of children with neonatal jaundice. In a study, maternal age was statistically significant with regard to the child’s serum bilirubin levels^21^. Mothers aged above or equal to 30 years constituted a greater risk for the development of jaundice in newborns^22^.

This study has limitations due to its retrospective nature, based on reports provided by mothers, which may be affected by memory bias. Thus, the lack of objective data, such as laboratory tests that could provide an objective diagnosis, demands caution in the interpretation of our results. Furthermore, the cross-sectional design, although it involves a large sample, can only point to associations and not to a causal relationship, which demands other studies with longitudinal methodology. However, the maternal role in the search for early diagnosis as an initial step for appropriate investigation and consequent success in jaundice management or treatment cannot be neglected. Therefore, the factors found in this study can contribute to the understanding of jaundice in newborns and help develop management methods to prevent the condition.

We can conclude that the jaundice rate of mother-reported newborns was 17.9%, which is associated with variables related to maternal and birth aspects. This finding can contribute to the development of primary care conduct and public policies since hyperbilirubinemia is involved in different important biological processes.
